# Intraoperative characterization of cardiac tissue: the potential of light scattering spectroscopy

**DOI:** 10.1117/1.JBO.29.6.066005

**Published:** 2024-06-05

**Authors:** Brian Cottle, Sarthak Tiwari, Aditya Kaza, Frank B. Sachse, Robert Hitchcock

**Affiliations:** a University of Utah, Department of Biomedical Engineering, Salt Lake City, Utah, United States; b Boston Children’s Hospital, Boston, Massachusetts, United States

**Keywords:** light scattering spectroscopy, machine learning, congenital heart defects

## Abstract

**Significance:**

Damage to the cardiac conduction system remains one of the most significant risks associated with surgical interventions to correct congenital heart disease. This work demonstrates how light-scattering spectroscopy (LSS) can be used to non-destructively characterize cardiac tissue regions.

**Aim:**

To present an approach for associating tissue composition information with location-specific LSS data and further evaluate an LSS and machine learning system as a method for non-destructive tissue characterization.

**Approach:**

A custom LSS probe was used to gather spectral data from locations across 14 excised human pediatric nodal tissue samples (8 sinus nodes, 6 atrioventricular nodes). The LSS spectra were used to train linear and neural-network-based regressor models to predict tissue composition characteristics derived from the 3D models.

**Results:**

Nodal tissue region nuclear densities were reported. A linear model trained to regress nuclear density from spectra achieved a prediction r-squared of 0.64 and a concordance correlation coefficient of 0.78.

**Conclusions:**

These methods build on previous studies suggesting that LSS measurements combined with machine learning signal processing can provide clinically relevant cardiac tissue composition.

## 1 Introduction

Over 40,000 children are born annually with congenital heart defects (CHDs). One-quarter of them will require surgery to correct their CHD within the first year of their lifetime.^1^^–^^3^ Damage to the cardiac conduction system (CCS), which includes the sinus node (SN), atrioventricular node (AVN), bundle of His, and left and right bundle branches, remains one of the most significant risks associated with these surgeries.^4^^,^^5^ While surgical methods of repairing CHDs have improved steadily since their introduction in the mid 20th century, iatrogenic damage to the CCS requiring permanent pacemaker implantation occurs in up to 26% of complex open-heart surgical procedures.^6^ The placement of a permanent pacemaker, though a life-preserving intervention, imposes significant and long-lasting physical and financial burdens on the patient and their families.^7^

The current best practice for avoiding damage to the CCS during surgery involves using anatomical landmarks, such as the triangle of Koch, coronary sinus, terminal crest, and cavoatrial junction to approximate the locations of the CCS and their supporting vasculature. This practice is based on pioneering research on the CCS performed in the 1800s and has been refined using modern histological methods.^8^^,^^9^ Though the use of superficial anatomical landmarks provides a solid basis for approximating the locations of CCS components and related supporting structures, recent studies have described significant variation in the location of these supporting structures in relation to their respective anatomical landmarks.^10^^–^^12^ It is also understood that the nodal tissues’ location varies significantly with respect to anatomical landmarks in hearts with CHDs.^9^^,^^13^^–^^15^ To account for these variations during surgery, surgeons proceed with greater caution around the “danger areas” where not only the CCS and supporting structures are thought to reside but also where they could potentially reside. This greater caution taken by surgeons results in an increased number of residual lesions, which levy their burden on patient recovery, resulting in poorer outcomes.^6^^,^^16^^,^^17^ Improving intraoperative localization of the CCS could reduce permanent pacemaker implantations while increasing surgeons’ confidence, thereby reducing the number of residual lesions. Whether through a decreased likelihood of pacemaker implantation or fewer residual lesions, improved CCS identification can positively affect patient outcomes.

Though numerous methodologies have been explored for intraoperative CCS identification, recent developments in approaches termed “optical biopsies” have shown promise to address the shortcomings of current approaches.^6^ Optical biopsies leverage the interactions between light waves and tissue to characterize tissue regions non-destructively. These tissue-light interactions are quantified through various optical methods, such as spectroscopy, fluorescence microscopy, and tomography. Due to their non-destructive and non-invasive characteristics, CCS localization approaches that leverage optical biopsy methodologies could provide actionable insights to surgeons both before in the case of a catheterized probe, and during surgical intervention.

Two optical biopsy approaches currently being explored to guide surgical procedures in the heart are fiber-optic confocal microscopy (FCM) and optical coherence tomography (OCT). Though these approaches have shown promise due to their comparatively high imaging resolution and ability to discern different tissue types within the heart, including nodal tissue regions, the significant financial costs, and complexity associated with their use have hindered widespread implementation.^18^^–^^20^

Optical methods that leverage spectroscopic measurement of light-tissue interactions have the potential to greatly decrease the implementation cost and complexity of FCM and OCT while overcoming the prohibitively small maximum imaging depths of current CCS identification methods.^21^^,^^22^ Previous research performed by our group has provided proof-of-concept of the imaging capabilities of our custom light-scattering spectroscopy (LSS) probe. LSS, a well-established optical measurement method used in various biological clinical and research settings, measures how different wavelengths of light scatter when traveling through a medium containing discrete particles.^23^^,^^24^ The LSS probe leverages the light-scattering properties of cardiac tissue in conjunction with machine learning and signal processing methods to perform tissue characterization and quantification using scattered light measurements.

Our custom-designed LSS probe, when coupled with machine learning (ML) signal processing methods, is capable of characterizing tissue properties such as the depth and arrangement of fibrotic tissue, the volume fraction of fibrotic tissue, and the density of nuclei within a tissue sample.^25^^,^^26^ While these studies provide valuable insight into the LSS-ML system capabilities, the studies were limited in scope as the tissue samples used for imaging represented simulated heterogeneity constructed from separate, homogenous samples of fibrotic and myocardial tissue. The tissue samples involved in these studies were also gathered from animal models, further limiting their relevance to the application of this technology in humans. To further validate and explore the capabilities of LSS combined with ML, this study aims to build upon the findings of these previous studies using the LSS probe to image the heterogenous CCS tissue regions from neonatal human hearts. We then correlate these LSS spectral measurements with the ground-truth composition of the imaged tissue samples by recreating 3D models of the excised tissue regions using an established method created previously by our research group. We then applied linear and non-linear machine learning methods to analyze and produce predictions from the dataset. We evaluated the predictions on holdout test datasets using a leave-one-out cross-validation (LOOCV) approach.

## 2 Materials and Methods

### 2.1 Tissue Excision, Sample Processing, and 3D Model Creation

Tissue samples were extracted, prepared, sectioned, and histologically stained following established protocols from Refs. 27 and 28, and reconstructed 3D models from Ref. 28 were used in this study. In summary of the methods described in Ref. 28, the tissue samples were excised, sectioned into 4  μm thick sections spaced approximately 25  μm apart and stained using an automated Masson’s Trichrome staining method. The sections were imaged on an automated slide scanner at a 0.44  μm/pixel resolution. The resulting images were registered and stacked to create serial images of the section tissue samples.

**Fig. 1:**
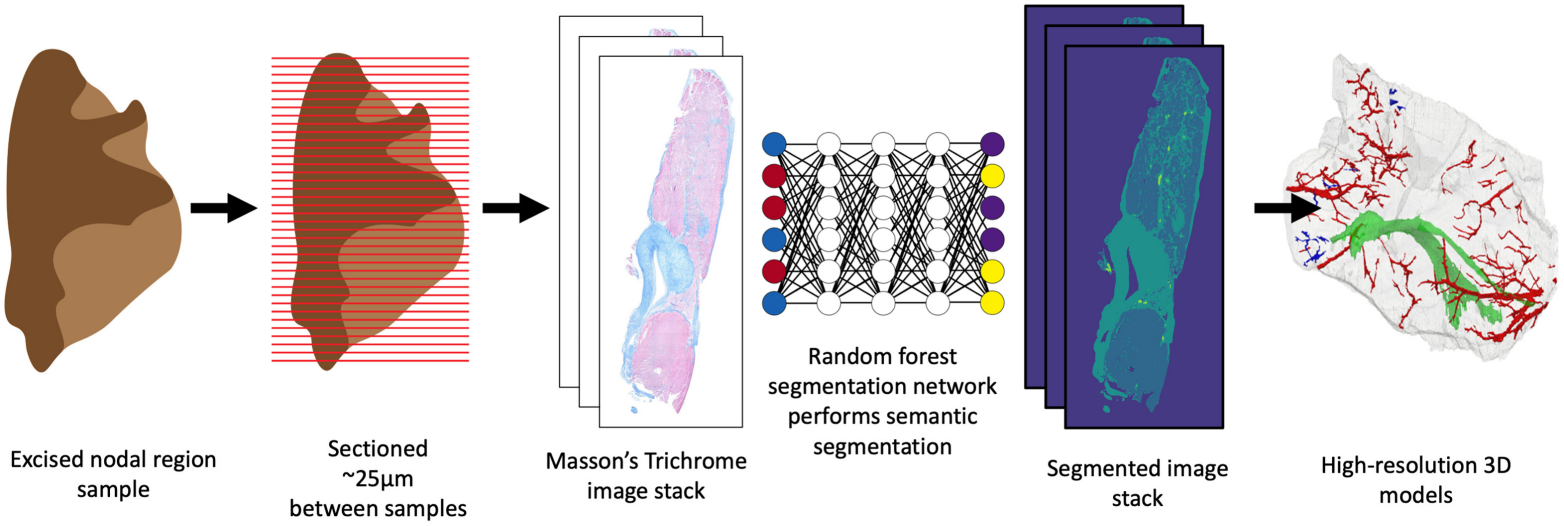
Pipeline used to create 3D reconstructions from excised cardiac tissue samples containing nodal tissue regions.

These serial images were then segmented using a random-forest segmentation network created in the software Ilastik.^29^ Most of the technical aspects of random forest parameter optimization, such as the number of trees, nodes, and other hyperparameters, were managed by the Ilastik software. For the model used in this work, the default value of 100 trees was used. The ground truth segmentations used for training the segmentation classifier were created in the Ilastik software using the provided image annotation tools. Forty images sampled randomly from 7 different nodal tissue sections were included in the ground truth segmentations used to train the random forest network. The model was trained to segment four different classes including myocardium, connective tissue, and separate classes for nuclei within the myocardium and connective tissue regions. As described in Ref. 28, the segmentation results were inspected and verified by an external expert cardiac pathologist. The details of post-processing the segmentation results to refine the nuclear segmentation are described in a later section. These segmented images were then used to create 3D reconstructions of the excised tissue samples. The average distance between slides was calculated using methods outlined in Ref. 28, and this distance was used to interpolate values for the connective and myocardial tissue samples between histological sections in the direction of sectioning using methods outlined by Schenk et al.^30^ The final voxel size of the 3D tissue composition model used for this work was 0.44  μm×0.44  μm×7  μm, where the third dimension is the direction of sectioning and interpolation. Figure 1 provides an overview of this process.

### 2.2 Tissue Prep for Data Acquisition

Following extraction of the nodal tissue regions of interest and before tissue sectioning and histological staining, both the sinus and AV nodal region samples had three fiducial markers placed transmurally on the epicardial surface for the sinus nodal regions and through the membranous septum and ventricular septum for the AV nodal regions. These fiducial markers were created using a 1.0 mm diameter disposable biopsy punch (Rapid-Core 1.0, World Precision Instruments, Sarasota, Florida, United States).

In preparation for LSS spectra acquisition, each tissue sample was placed onto and pinned to a thin sheet of open-pore black foam, preventing the backscattering of photons after passing through the sample. The well dish was then filled with 1× phosphate buffered solution to the point of submersion of the tissue within the solution.

**Fig. 2:**
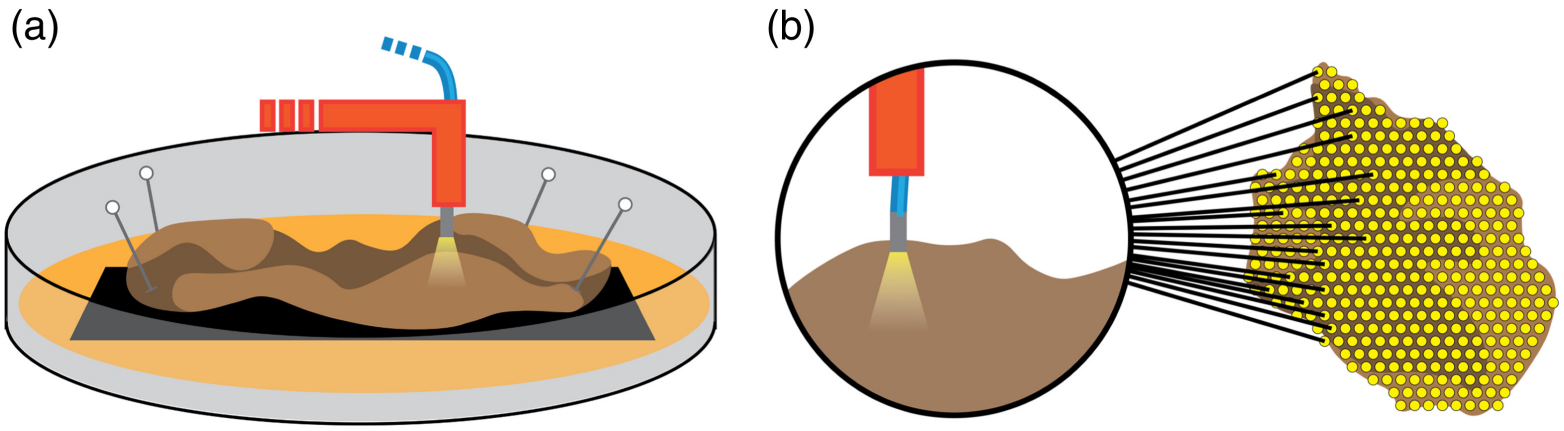
Placement of the custom LSS probe in a fixture and used to gather spectral data in an orientation normal to the surface of the tissue. (a) Placement of the tissue onto a black open-pore foam within a well-dish. (b) Example grid pattern created and used to gather data across the surface of the tissue. Yellow spots indicate individual, unique spectral sampling locations.

The custom-designed LSS probe was connected to a tungsten-halogen broad-spectrum light source (SLS201L/M, Thorlabs, Newton, New Jersey, United States), which emitted light in the 350 to 2000 nm range and two Czerny-Turner type charge-coupled diode (CCD) spectrometers (CCS175/M, Thorlabs). The spectrometers were also connected to a computer running custom spectrum analysis software (Cite LSS Nuclear paper). The tip of the LSS probe was then attached with a custom fixture, holding the probe normal to the workbench’s surface and pointing down. The fixture was then attached to a three-axis micromanipulator (MP-285, Sutter Instrument Company, Novato, California, United States) magnetically fixed to the workbench. The well dish containing the excised tissue sample was placed directly beneath the LSS probe tip and aligned with markings on the workbench for consistent placement and orientation. SN samples were oriented by placing the epicardial surface facing up, with the superior vena caval aspect toward the left of the acquisition setup and the atrial appendage aspect toward the right of the setup. AV nodal tissue samples were oriented with the right atrial aspect facing up, the membranous septum aspect toward the top of the acquisition setup, and the ventricular septal aspect toward the bottom of the setup. The acquisition setup’s top/bottom and left/right orientations correspond to the x and y axes of the micromanipulator fixture, respectively. An illustration of this setup can be found in Fig. 2(a).

Custom software written in MATLAB (R2022b, MathWorks, Natick, Massachusetts, United States) was used to control the micromanipulator and extract spectral measurements from the two spectrometers attached to the LSS probe. This software also recorded the location in 3D space of where each spectral measurement was taken.

### 2.3 LSS Data Acquisition

The 3D locations of the three fiducial marks in the tissue were acquired by using the micromanipulator to position the LSS probe tip concentric with the fiducial mark above the tissue, and then lowering the tip of the probe to contact the surface of the tissue and recording the tip location. Each fiducial mark location was sampled three times, and the probe tip was moved away from the tissue between each measurement before performing a subsequent measurement.

After recording the locations of the fiducial marks, LSS data acquisition locations were mapped across the surface of the tissue sample in a diamond grid pattern, with 280  μm between every individual sample location across the tissue surface [Fig. 2(b)]. A trained operator confirmed sufficient contact of the probe tip with the tissue surface at each grid location. After confirming the contact of each grid position with the tissue surface, automated acquisition of spectral samples using the LSS probe was initiated within the custom MATLAB software.

Three hundred spectral samples were acquired at each grid location. Each spectrum was sampled with an integration time of 70 ms and a full width at half maximum resolution of 0.6 nm within the 500 to 1000 nm wavelength range. Upon measuring the 300 spectra, their values at each wavelength were averaged and stored as a single spectral measurement associated with the 3D location at the grid point.

After the topographical spectral measurements were completed, the 3D locations of each of the three fiducial markers were recorded following the same protocol as before grid sampling. The grid locations and spectral data were resampled if any drift or notable change was noted between the initial fiducial mark locations and the post-spectral acquisition locations.

Once the tissue samples were fully sectioned, stained, imaged, and digitally processed, a manual segmentation process was used to identify the location of the fiducial markers within the tissue samples using the image processing software FIJI.^31^ These segmentations were then processed following the same protocol as the myocardial and connective tissue segmentations to create 3D volumes for each fiducial marker in the tissue samples.

### 2.4 Nuclear Segmentation Verification

The Ilastik-based nuclear segmentation was validated on serial histological sections of ovine cardiac tissue. The samples were acquired, excised, sectioned, and stained following the protocols outlined by Johnson et al.^27^ Four serial section pairs of tissue, all acquired within 400  μm of each other from within the tissue block, were identified for nuclear segmentation validation. One section from each pair was stained, imaged, and segmented using the Masson’s trichrome-based segmentation described previously. The other tissue section from each pair was stained using a DAPI staining medium (#D1306, ThermoFisher Scientific, Waltham, Massachusetts, United States) and imaged using the same automated slide scanner (Carl Zeiss AG, Oberkochen, Germany). DAPI nuclear segmentation and post-processing image refinement involved thresholding the DAPI image at two standard deviations above the median intensity value, applying a water-shedding algorithm to separate the nuclei, and filtering out segmented objects that were smaller than $20\;{\mu m}^{2}$ and larger than $100\;{\mu m}^{2}$. Nuclear densities within and without the segmented nodal regions were calculated for both sinus and AV nodal regions. Nodal tissue segmentations were performed according to methods outlined in Ref. 28. A trained cardiac pathologist validated segmentations of other tissues, including connective, myocardial, and nodal tissues.

### 2.5 3D Model Exclusion Criteria

After the stained tissue slides were prepared, tissue samples were excluded from the analysis if they contained at least five slides that were stained insufficiently. Insufficient staining is defined in this context as including inconsistent and abnormally light or heavy staining across the tissue section. Additionally, tissue sections were excluded from subsequent analysis if more than five slides contained extensive damage to the tissue or excessive inflammation or calcification.

### 2.6 LSS Point Registration Methods

Registration of the LSS probe grid locations to the surface of the 3D tissue model was performed by registering a minimum of 10 paired points corresponding to the fiducial marks and other landmarks within the 3D model and the probe grid. The locations of the fiducial markers were identified as the centroids of the segmented 3D volumes associated with each fiducial mark placed in the tissue samples. A transformation matrix was found by optimizing the Procrustes distances between the paired points.^32^^,^^33^ The average distance between serial tissue sections was determined by altering the distance between sections and optimizing the registration between the probe grid fiducial marker locations and their corresponding paired points along the surface of the 3D tissue model. These methods resulted in applying a similarity transform to the LSS data points, associating the spectra to a registered location on the tissue surface of a 3D reconstructed tissue model.

### 2.7 Volume Composition Information Extraction

After registering the probe grid locations onto the surface of the 3D tissue models, tissue composition information was extracted from the 3D models at each point within the LSS probe grid. A conical frustum volume was used to determine the 3D volume of interest associated with each point in the LSS probe location grid. The top diameter of the frustum was set to 540  μm, which is the maximum distance between sensing fibers at the tip of the LSS probe. The angle of the sides of the frustum was set to the full acceptance angle, also called the divergence angle, of the sensing fibers, 12.71 deg, which was calculated using Eq. (1) using a numerical aperture (NA) of 0.22 for the silica optical fiber (FVP100110125, Molex)

$$\frac{\sin^{-1}(NA)}{2}=\text{acceptance angle}.$$ (1)

**Fig. 3:**
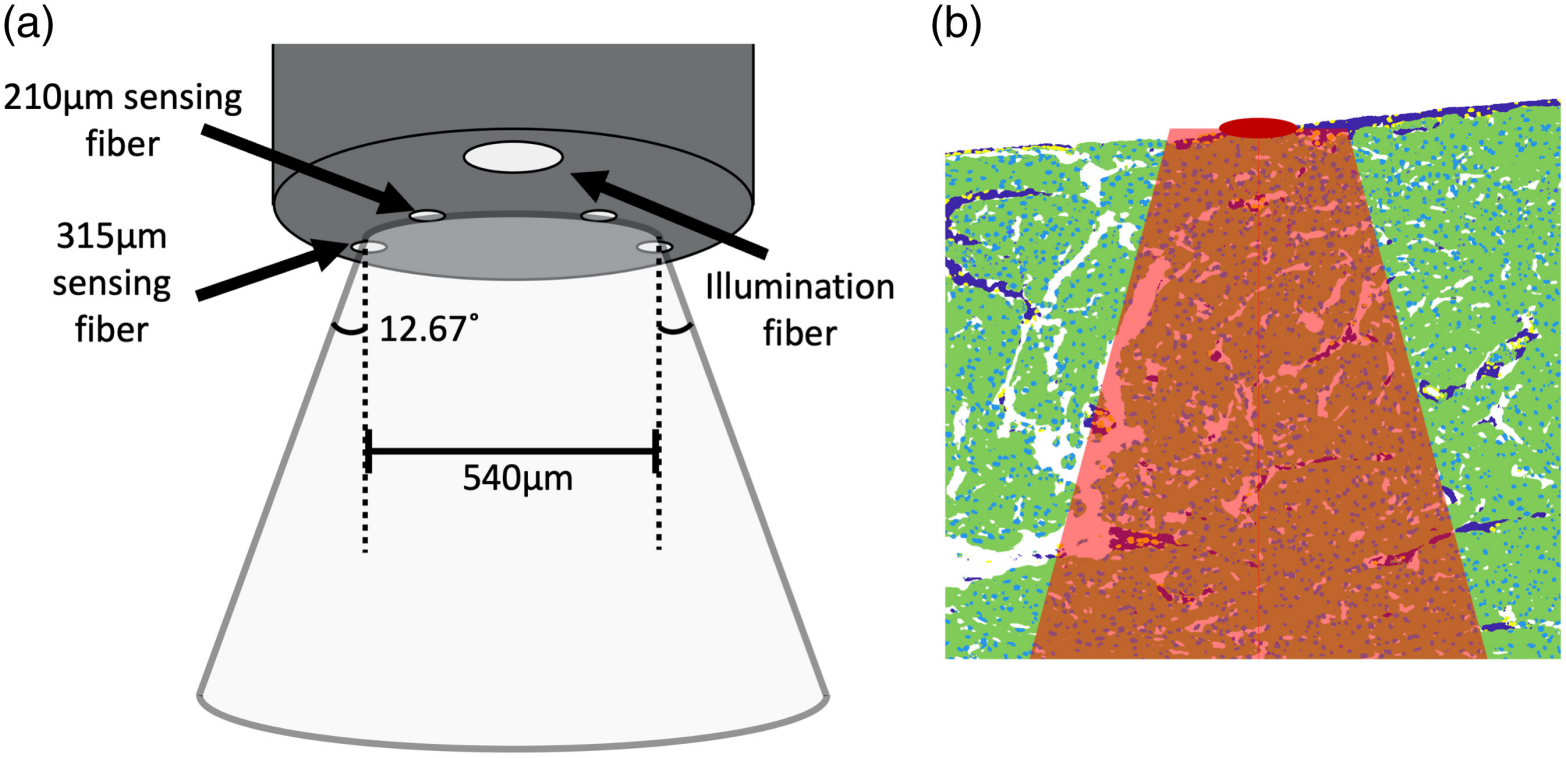
Relationship between the tip of the LSS probe, calculated frustum, and segmented tissue models. (a) Probe tip describing how the sensing fibers relate to the initial diameter of the frustum, as well as how the angle calculated from the numerical aperture of the fibers relates to the increasing diameter of the frustum as a function of distance from the probe tip. (b) Cross-section of segmented tissue. Dark blue indicates segmented connective tissue, green indicates myocardium, light blue and yellow indicate segmented nuclei; dark red indicates the center of the location where the probe was placed; and the light red overlay indicates the region, based on the frustum that would be included in the analysis for the given point on the tissue surface.

Figure 3(a) shows how the frustum relates to the tip of the custom LSS probe. The frustum was then oriented in 3D space such that the top surface was positioned at a given 3D location in the probe grid concentric with the orientation of the LSS probe and the Z axis. The frustum volume extended transmurally through the tissue, containing tissue spanning the entire depth of the tissue sample directly below the given location. An example of how this extends through segmented tissue can be seen in Fig. 3(b). All 3D model voxels contained within the frustum were included in the tissue composition measurements, and statistics, such as nuclei per μm and muscle volume fraction, were associated with the spectral measurements acquired at the given point. All image processing and association of LSS spectra with tissue composition information were performed using custom software written in MATLAB.

### 2.8 Preprocessing the LSS Signals

All LSS spectra were calibrated using a spectrum from a 99% reflectance factor white diffuse standard reference (Spectralon, Labsphere Inc., North Sutton, New Hampshire, United States). Calibration spectra were re-acquired before measuring LSS data from each tissue sample. These individual calibration spectra were used only with the LSS spectra from the associated tissue sample. The spectra were then individually z-scored and grouped according to the tissue sample. Mean and standard deviations used in z-scoring were calculated within each tissue sample group of spectra. After preprocessing, the spectra from the 210 and 315  μm sensing fibers for each measurement location were concatenated to facilitate one-dimensional input into the linear regression methods used in this study. All preprocessing of spectra and training of regression models was performed in custom code written in the Python programming language.

A principal component analysis was performed on the LSS spectra by extracting the principal components from the spectra and visualizing the first two components and their association with the nuclear density and muscle volume fractions. Additionally, dimensionality reduction was also performed using the non-linear uniform manifold approximation and projection (UMAP) method.^34^

### 2.9 Train-Test Split and Leave-One-Out Cross-Validation

The calibrated, z-scored, and concatenated spectra were then used for training both linear regression models and multi-layer perceptron regressors to predict nuclear density in nuclei per $\mu m^{3}$ and muscle or connective tissue volume fraction as a percent of the volume of the extracted frustum. After preprocessing, the input for each model was a collection of calibrated, z-scored spectra acquired from the custom LSS probe. The output or ground truth associated with each individual spectra was either a nuclear density value or a fraction. To facilitate training and model convergence, both ground truth measurements were scaled to a range between 0 and 1000 to improve convergence while training linear and neural network-based regression methods.

The data were split into training and testing datasets following an LOOCV method.^35^ This method entailed creating a training dataset that included all the spectra minus the spectra and data associated with a single tissue sample. This “hold out” set of data, which consisted of all the spectra associated with the given tissue sample, was then used to test both the linear and non-linear regressor models trained on the given training dataset. A total of 14 train-test splits were created using this method, where each test set contained all the spectra associated with a given, unique tissue sample that was excluded from the training set in the given test-training split.

### 2.10 Linear Approximation Methods

The processed spectra were then used, in conjunction with the scaled muscle volume fraction and nuclear density ground truth values, to train a linear regressor model. The model was trained in Python using the scikit-learn package. A linear regressor using L1 and L2 data priors as regularization parameters, also called an Elastic Net,^36^ was trained and evaluated using the LOOCV approach described earlier. A small hyperparameter search was carried out and determined that an alpha regularization coefficient of 1.0, with a maximum number of iterations of 100 produced the most stable, consistent results throughout the cross-validation. A new linear regressor model was trained for each of the 14 train-test splits in the cross-validation. Default values were kept for the L1 ratio, and alpha parameters were used for training. The results from the individual test predictions were aggregated and evaluated using r-squared and concordance correlation coefficients.

### 2.11 Weight Analysis of Linear Models

The input weights of the linear model were collected from each training session within the cross-validation. The weights were then averaged across the training sessions and visualized for inspection to determine their relative importance in the outcome of the regression analysis.

### 2.12 Neural Network Analysis

A small, fully connected neural network was created and trained as a regressor model to predict muscle volume fraction and nuclear density from the processed spectra. This network consisted of an input layer and two fully connected layers, each of which was subsequently followed by a batch normalization and dropout layer. A hyperparameter search that included the dropout percentage and number of nodes in each fully connected layer was carried out, resulting in a final network topology with values of 35 and 0.2 for the number of nodes and dropout percentage, respectively.

The neural networks were trained and evaluated following the same LOOCV method as the linear model. A new model was trained for each of the 13 train-test splits in the cross-validation. The networks were trained for a total of 40 epochs with a learning rate of 0.1.

## 3 Results

**Fig. 4:**
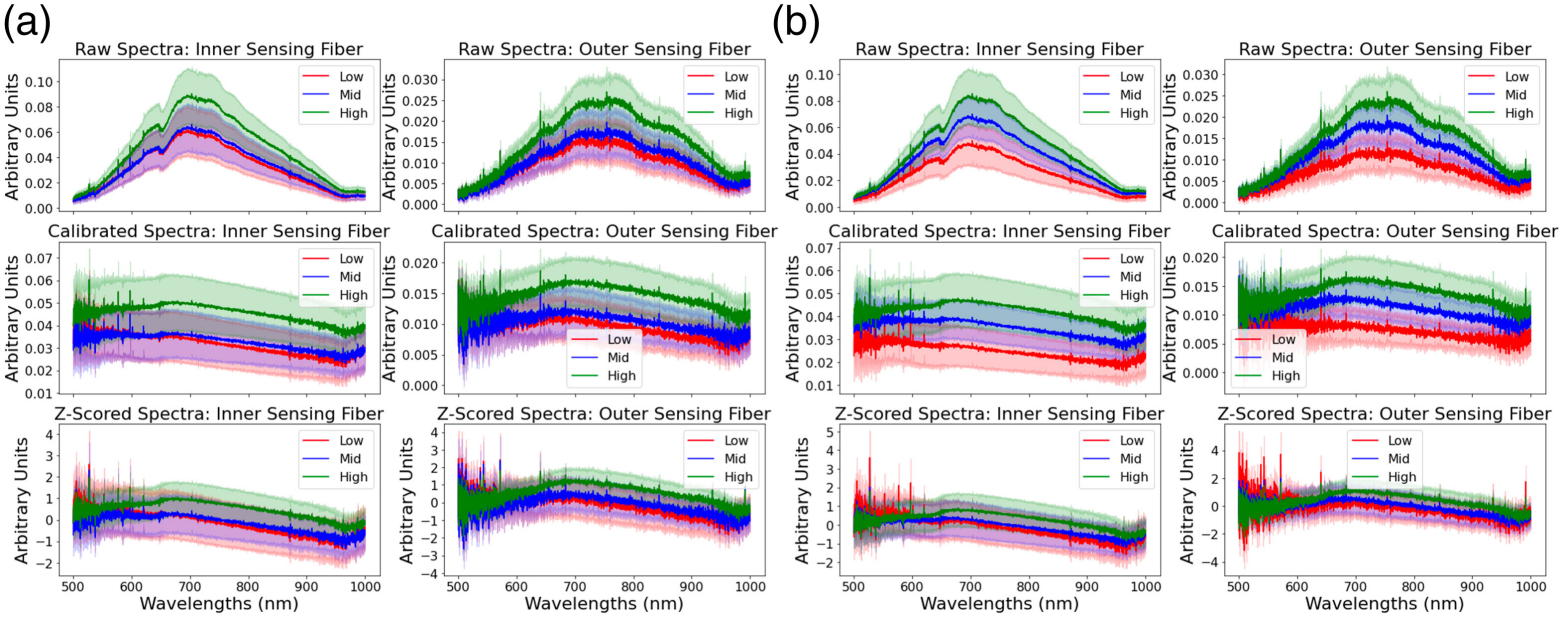
Distributions of spectra for the inner (210  μm) and outer (315  μm) sensing fibers across the major preprocessing steps, showing raw, calibrated, and z-scored spectra. Spectra are grouped according to low (0’th to 20’th percentile), mid (40’th to 60’th percentile), and high (80’th to 100’th percentile) for both the muscle volume fraction (a) and nuclear density (b).

Nine sinus and 10 AV nodal regions were excised and processed according to methods outlined in Ref. 28. After applying exclusion criteria to the final 3D reconstructions and histological images, a total of eight sinus and six AV nodal tissue regions were included in the analysis. In sum, light-scattering spectra were gathered from approximately 13,000 unique locations across all the tissue samples included in the analysis. The distributions of these spectra are shown in Fig. 4, as well as demonstrations of how the preprocessing pipeline alters the spectra.

**Fig. 5:**
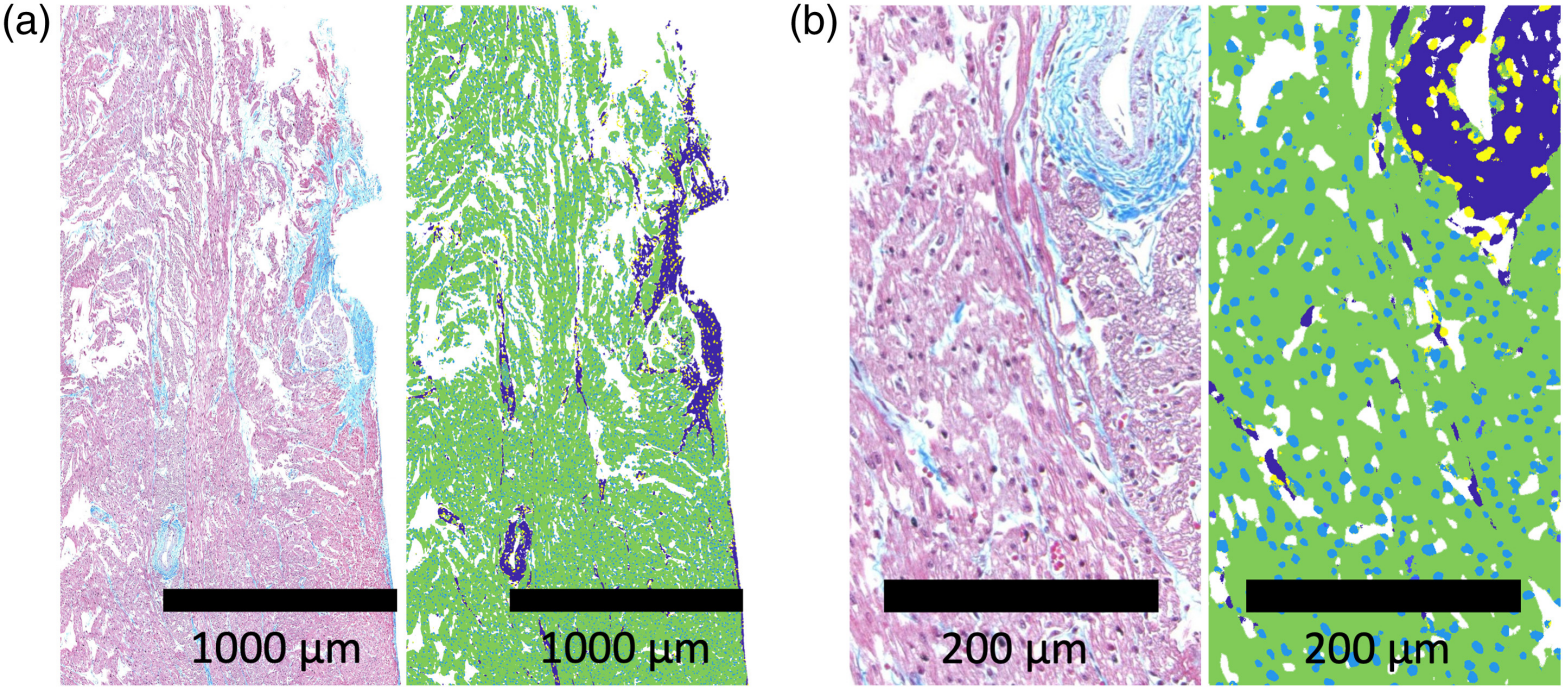
Example segmentations from the random-forest segmentation network. (a) Higher-level overview of a cross section of the ventricular septum. (b) Higher resolution view of the same region. Dark blue indicates connective tissue, green indicates myocardium, light blue indicates myocyte nuclei, and yellow indicates fibroblast nuclei.

Approximately 4000 serial histological images were processed and included in the reconstruction of tissue samples included in our analysis. The segmentations of these tissue samples, which include segmentations of the nodal regions, myocardium, connective tissue, and nuclei, were scrutinized and validated by an expert clinical cardiac pathologist. Examples of these images and their segmentations are shown in Fig. 5.

### 3.1 Nuclear Segmentation Validation

The average area of nuclei segmented from DAPI-stained reference tissue images was $43.55\pm 18.96\;{\mu m}^{2}$. The average area of nuclei segmented using the random-forest segmentation algorithm on Masson’s trichrome-stained slides was $36.03\pm 14.1\;{\mu m}^{2}$. The mean number of nuclei counted on the DAPI reference slides was 91,807±12,499 nuclei, with the mean number of nuclei counted on the Masson’s slides being 91,116.63±9877 nuclei. The difference between the two as a percentage of the count from the DAPI reference slides was less than 1%.

The nuclear density within the sinus nodal regions of the tissue samples was found to be $5.3•10^{-5}\text{ nuclei}/{\mu m}^{3}$. This was found to be greater than the surrounding tissues (P≪0.05), which had an average nuclear density of $3.7•10^{-5}\text{ nuclei}/{\mu m}^{3}$. The nuclear density within the AV nodal regions was $6.24•10^{-5}\text{ nuclei}/{\mu m}^{3}$, which was found to be less than the density of the surrounding tissues at $7.87•10^{-5}\text{ nuclei}/{\mu m}^{3}$ (P<0.05).

### 3.2 Dimensionality Reduction Analysis

**Fig. 6:**
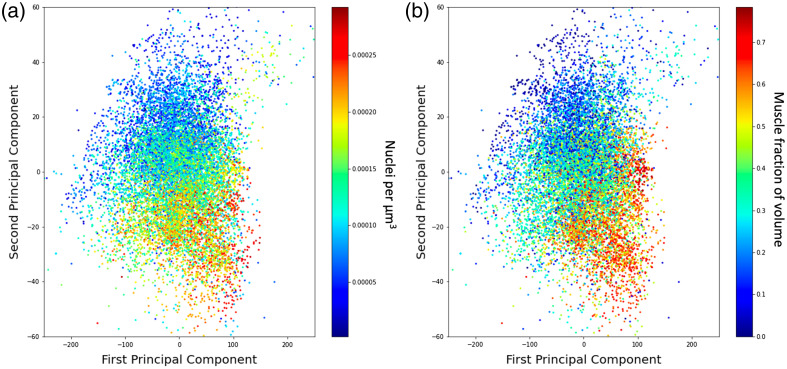
Principal components and associated tissue composition features. (a) Correlation of the nuclear density with the first two principal components. (b) Correlation muscle volume fraction with the first two principal components.

Visualizations of the first two principal components of the spectral data can be seen in Fig. 6. This principal component analysis demonstrates the correlation between the nuclear density and the spectral data [Fig. 6(a)] and the muscle volume fraction and the spectral data [Fig. 6(b)] as a gradient increasing toward the bottom right of the plot. The results from the UMAP analysis demonstrated greater sensitivity to the batch effect, clustered the spectra more according to tissue sample, and demonstrated less correlation with the nuclear density and the muscle volume fraction.

### 3.3 Linear Regression Analysis

**Fig. 7:**
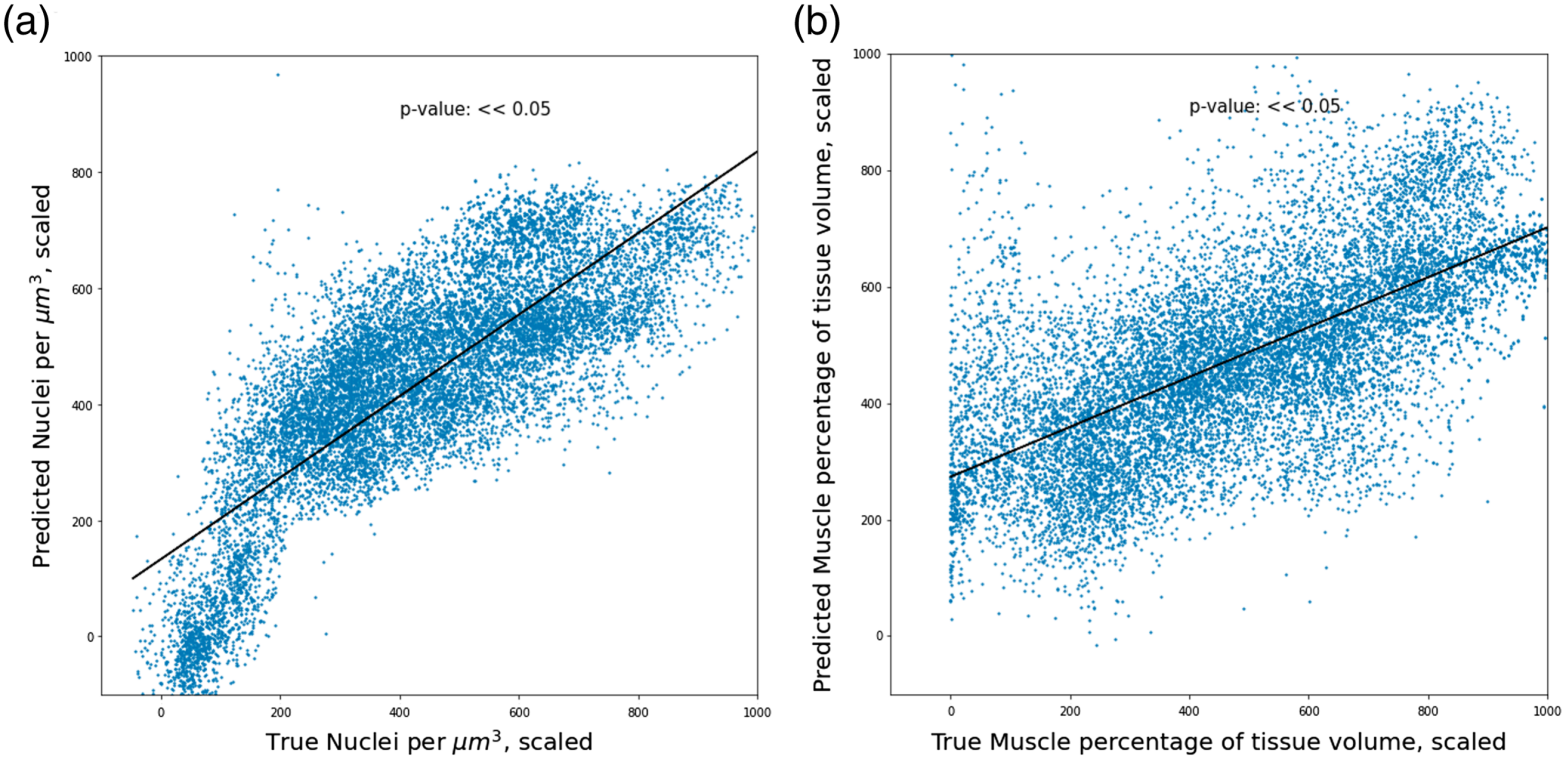
Relation between predicted values and ground truth resulting from the elasticnet regressor via the aggregated LOOCV prediction results. (a) Results from the prediction of nuclear density. (b) Results from the prediction of muscle volume fraction.

A total of 14 elastic net regressor models were trained following the LOOCV approach. The nuclear density regressor achieved a prediction r-squared value of 0.64 and a concordance correlation coefficient of 0.78. The muscle volume fraction regressor achieved a prediction r-squared of 0.41 and a concordance correlation coefficient of 0.59. Figure 7 shows the aggregated test-set predictions of the individual regressor networks across the cross-validation.

**Fig. 8:**
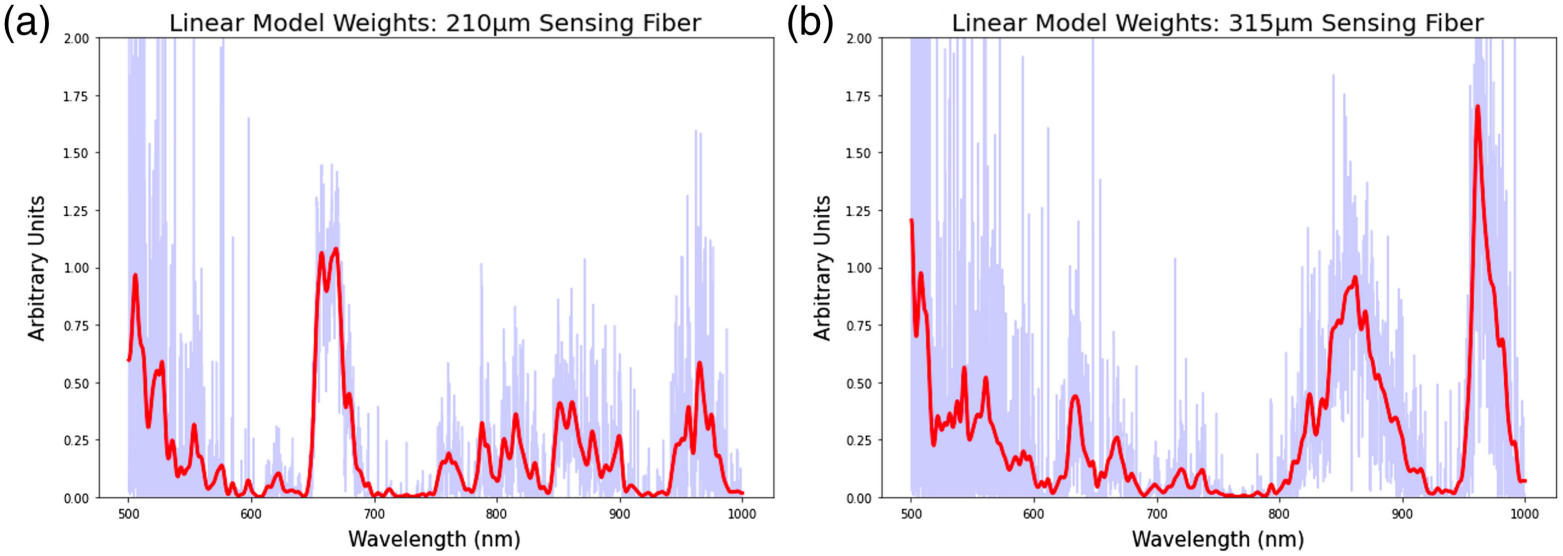
Elasticnet linear model weights aggregated and averaged across all LOOCV training sessions associated with the nuclear density. The light blue trace shows raw weight values, and the red trace indicates the weights after a smoothing function was applied. (a) Weight values per wavelength for the sensing fiber 210  μm from the illumination fiber. (b) Weight values per wavelength for the fiber that is 315  μm from the illumination fiber.

Figure 8 shows the weights of the nuclear density elastic net regressors for both the 215  μm sensing fibers and the 310  μm sensing fibers. This analysis indicates that the nuclear density regressors weighted wavelengths below 600 nm and wavelengths between 650 and 700 nm highly for spectra acquired from the 215 nm sensing fiber. The nuclear density regressors also weighted wavelengths below 600 nm highly for measurements from the 310 nm sensing fiber but weighted other frequency bands differently, including between 800 and 900 nm as well as 950 to 1000 nm.

**Fig. 9:**
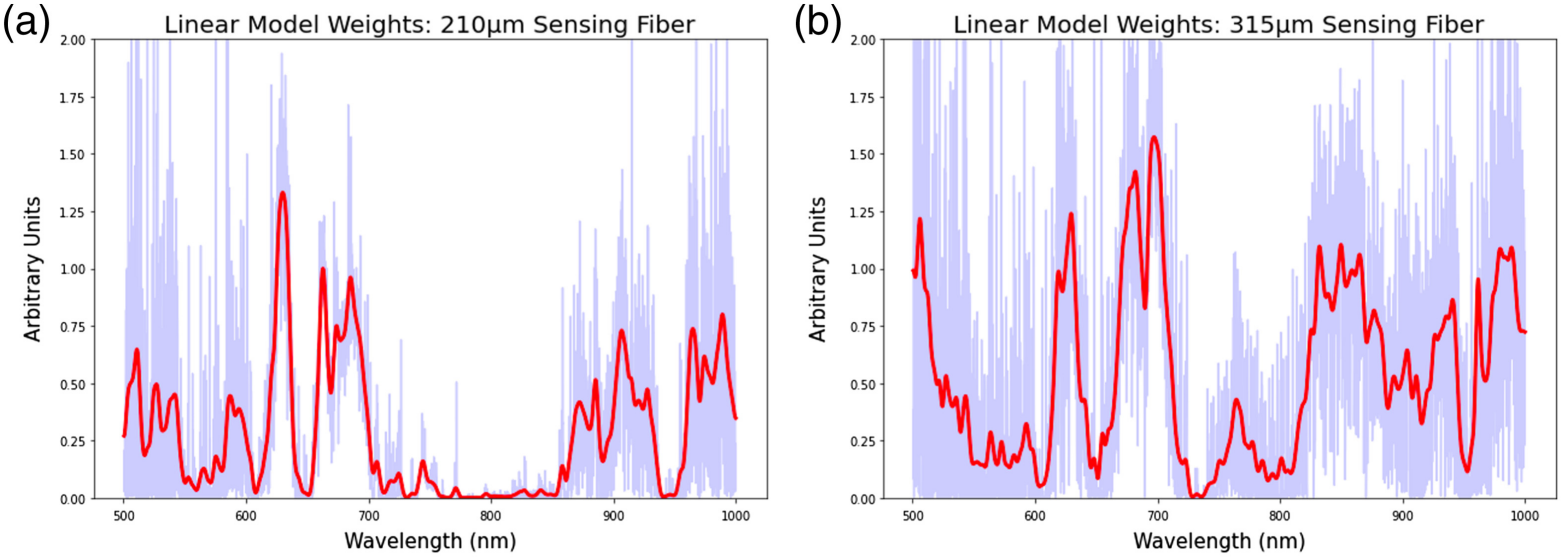
Elasticnet linear model weights aggregated and averaged across all LOOCV training sessions associated with the nuclear density. (a) Weight values per wavelength for the sensing fiber 210  μm from the illumination fiber. (b) Weight values per wavelength for the fiber that is 315  μm from the illumination fiber.

Figure 9 shows the weights associated with the muscle volume fraction elasticnet regressors. This analysis indicated that, unlike the nuclear density regressors, the muscle volume fraction regressors applied a more similar weighting to both the 215 and 310  μm sensing fibers, placing higher weights on wavelengths between 600 and about 700 nm. The volume fraction regressors also placed higher weights on wavelengths greater than 850 nm for the 215  μm sensing fiber and greater than 800 nm for the 310  μm sensing fiber.

### 3.4 Neural Network Regression Analysis

Similar to the linear regression analysis, 14 fully connected neural networks were trained and tested following the LOOCV approach. The nuclear density regressor achieved an r-squared value of 0.62 and a concordance correlation coefficient of 0.79. The muscle volume fraction regressor achieves an r-squared of 0.41 and a concordance correlation coefficient of 0.63, producing results nearly identical to those from the linear regressor.

## 4 Discussion and Conclusion

This study presents a novel pipeline for gathering LSS measurements from tissue samples and correlating those measurements with ground truth composition metrics gathered from 3D reconstructions of excised tissue samples. Numerous studies have been carried out exploring the benefits of using LSS for tissue characterization, including cancer diagnosis and the evaluation of polyps and ulcers.^24^^,^^37^

Similar technologies have been explored for use during surgery, such as OCT, confocal microscopy, and spectroscopy methods, such as reflectance and Raman spectroscopy.^38^^,^^39^ Fiber optics confocal microscopy, as one example, has been used during open heart surgery to distinguish tissue regions.^18^ However, due to significant financial costs and complexity associated with refining their use and deploying these technologies in the operating room, they have yet to see widespread adoption.

There are visible differences in the spectra associated with high and low nuclear densities and volume fractions. These differences are noted across the major spectral preprocessing steps (Fig. 4). Calibration of the spectra was performed to reduce the amount of information retained in the spectra associated with the individual spectrometers. As two individual spectrometers were used in the analysis, each spectrometer was consistently associated with only one of the sensing fibers. For example, spectrometer 1 was consistently used to gather data from the inner sensing fiber, and spectrometer 2 was used to gather data from the outer sensing fiber. The calibration approach largely eliminated the unique baseline spectral waveform associated with each. These unique waveforms can largely be seen by comparing the top two plots in either Figs. 4(a) or 4(b), which show how the spectra acquired from the inner and outer sensing fiber differ. After calibration, these waveforms are largely suppressed, shown in the middle two plots of Figs. 4(a) and 4(b). Z-scoring on a per-feature basis was included in the pipeline to scale the spectra in a way that facilitated faster training and convergence of the models trained on the spectra. Although the z-scoring reduced some apparent separation, it was not always effective. For example, in the nuclear density [Fig. 4(b)], we found no significant difference in the final performance of models trained on the calibrated data versus models trained on the calibrated and z-scored data. Ultimately, though not providing a clear benefit and improvement to the evaluation metrics, z-scored data was used for all model training as a best practice approach for ensuring the input data for the regression analyses were all within an appropriate range.

LSS, when combined with machine learning, has shown promise as a potential cost-effective means of performing optical biopsies.^24^^,^^26^^,^^37^ This can be used to evaluate and predict tissue composition during open heart surgery. LSS is effective at quantifying nuclear density across homogenous tissue samples as well as quantifying and localizing fibrous tissue. These metrics are associated with physiological changes in cardiac tissue.^25^^,^^26^ The volume fractions of muscle and connective tissue have been well studied in the regions surrounding the SN and are known to be different than the composition of the SN.^40^^–^^42^ Additionally, portions of the AVN and other connected conduction system components are surrounded by or encapsulated by fibrous, collagenous tissue,^43^ increasing the contrast in tissue characteristics when comparing the AVN to the immediately surrounding tissues.

After validating our nuclear segmentation methods, we found that the nuclear density in the SN is higher than that of the surrounding tissue, which is a similar result to the nuclear densities found in studies carried out on rats.^44^ In contrast, however, the nuclear density of the AVN was found to be lower than the surrounding tissues. These findings, in conjunction with the understanding that muscle volume fraction is different between the nodes and surrounding tissues, support the hypothesis that quantifying cardiac tissue composition in real-time could lead to successful localization of the nodal regions. We also note that the average area of nuclei reported from both the Masson’s trichome and the DAPI stained images are likely underestimates, given the average area of the nucleus of a cardiomyocyte is typically between 50 and $80\;{\mu m}^{2}$.^45^ However, this underestimate is unlikely to affect the assessments of nuclear density and myocardial volume fraction.

The principal component plots in Fig. 6 show a correlation between the components and both the nuclear density and the muscle volume fraction. When visualizing the spectra using other non-linear dimensionality reduction methods, such as U-MAP,^34^ the spectra begin to cluster together in groups more closely associated with their respective tissue samples as opposed to other tissue composition-related features. This indicates that when training regression methods to predict tissue composition, the results could be sensitive to batch effects and overfitting, resulting in poor performance and overfitting for models with a high number of parameters. For this reason, we implemented low-complexity regression methods, including an elastic net linear regressor and a shallow fully connected neural network.

Using LOOCV as a method for evaluating model performance enabled us to minimize overfitting our datasets due to batch effects associated with each of the individual tissue samples. When analyzing the results of the aggregated LOOCV test datasets, the resultant r-squared values indicate a moderate correlation between the prediction values and the ground truth. To complement the test data r-squared measurement, we included an evaluation of the agreement between the ground truth and the predicted value, not just the correlation, by calculating the concordance correlation coefficient. These results also indicated reasonable agreement between the output of both the regression methods implemented and the ground truth nuclear densities and muscle volume fractions. The non-linear neural-network analysis prediction results differed very little from the linear elastic net regression, indicating that the relationship between the information gathered using the LSS probe and the tissue composition features likely does not require complex, non-linear, or more “black box” machine learning methods to perform predictions.

An analysis of the weights learned by the linear regressor model highlights how LSS can leverage the information associated with scattered photons as they travel through tissue substrates. The weights learned from predicting the nuclear density values differ slightly from those learned during the muscle volume fraction regression. In the nuclear density regression analysis, a difference was found between the weights learned on the 210  μm sensing fiber versus the 315  μm sensing fiber, where linear regressor placed emphasis on the 650 to 700 nm range in light acquired by the 210  μm fiber and placed lower emphasis on the 650 to 700 nm range and higher emphasis on wavelengths greater than 800 nm when gathered using the 315  μm fiber. This is likely due to a combination of the wavelength-dependent scattering properties of the aligned cylindrical structures in the myocardium^46^ and the fact that nuclei are one of the primary scatterers in biological tissue via wavelength-dependent mie scattering.^47^ This wavelength-dependent scattering due to nuclear density likely plays less of a role in the prediction of myocardial volume fraction, the information from which is more likely associated with other scatterers, such as the cylindrical and tube-like sarcomeres within the myocardium, hence the differences in spectral weights of the linear regressor.^47^ These wavelength dependencies could be used to inform future iterations of the custom LSS probe used in these experiments, as lasers of specific wavelengths could be used to increase the intensity of the light source, in turn reducing the amount of time required to acquire a sufficient spectral signal for analysis.

Studies have been carried out exploring the use of the anisotropic nature of fiber-oriented tissues, such as the myocardium.^46^^,^^48^ In this work, we focused on a broad spectrum of visible, unpolarized light, as our probe was designed to account for and neutralize the effects of polarization and anisotropic light-scattering behavior of tissue.^25^ In the future, however, should the specific relevant wavelengths be used to select lasers to provide light through the illumination fiber in the LSS probe, it could be beneficial to revisit the use and measurement of light scattering spectra via polarized light.

Future work would also benefit from approximating and accounting for the lens-like behaviors of the optical fibers. Additionally, we note that though the histological methods used in this study are the current gold standard for identification of the CCS in tissue samples, some deformation of the tissue occurs during the dehydration and embedding process in preparation for sectioning of the tissue. Our methods, however, do follow similar approaches when analyzing the sectioned and stained tissue samples in 3D, which provide precedent for the methods and analyses used in this study.^27^^,^^49^ Because our registration process for aligning the LSS data points to the surface of the tissue accounts for scaling between the two coordinate systems, we believe the results to be minimally affected by the deformation occurring due to histological staining. Future translation work involving nuclear density measurements, however, will need to account for differences in the volume between dehydrated tissue samples and *in vitro* tissues.

The use of a similarity transform when registering the LSS data points to their respective locations on the surface of the 3D tissue models has its limitations. During the reconstruction of the 3D tissue samples some models demonstrated a very slight sheering, which, when registered with the LSS locations, caused less than 1% of the LSS data points to be positioned slightly removed from the tissue surface. When this occurred, the LSS samples were excluded from the analysis. In future analyses a more robust registration approach, using affine transformations with a greater number fiducial markers, would improve the registration accuracy.

Future work could leverage the suitability of the 3D models generated in these experiments to Monte Carlo simulations, to further evaluate the propagation of light through the tissue regions of interest. Studies could leverage the data-rich nature of 3D models produced using these methods to perform both Monte Carlo simulations of propagations of different wavelengths through the tissues as well as ray tracing. These methods could add valuable insights to further inform the potential of optical methods when investigating and characterizing cardiac tissues. These simulations were not pursued in this study due to the complexity of developing an appropriate modeling approach to utilize the histology-based segmentations. Using similar segmentation approaches, a single-cell segmented dataset from the histological images coupled with ray-tracing or Monte Carlo simulations could add another additional dimension to similar analyses. We also note that the methods used for calculating the receiving volume, described in this paper as a frustum, were a significant approximation. Monte Carlo simulations would have enhanced this approach as well, allowing for a more precise approximation of both the receiving volume as well as appropriate penetration depths of photons.^50^^,^^51^

Due to the simple nature of the relationship between the acquired spectra and tissue features and the low cost associated with manufacturing the LSS probe, a simple device such as the one evaluated in this set of experiments could potentially be used as a means of inexpensively characterizing tissue properties during open-heart surgery. Due to the increased number of residual lesions associated with carefully avoiding cardiac conduction tissues, it is imperative that more precise methods of non-destructively characterizing cardiac conduction tissues be developed. Reliable intraoperative tissue characterization methods may enable the localization and precise identification of CCS components with fewer residual lesions. Our evaluation of the relationship between tissue characteristics and information gathered using light LSS further demonstrates the validity and potential of this technique as a means of inexpensively quantifying relevant cardiac tissue properties.

## Data Availability

The data produced in this study are available from the University of Utah Research Data Repository at the following link: [10.7278/S5d-1mxa-ffa0]. The code used in the analysis and preparation of the data for this article is available at the following GitHub repositories: https://github.com/brian-cottle/Histology-ML. https://github.com/brian-cottle/pediatric-heart-models
